# Rifabutin but not rifampicin can partly out-balance P-glycoprotein induction by concurrent P-glycoprotein inhibition through high affinity binding to the inhibitory site

**DOI:** 10.1007/s00204-023-03618-w

**Published:** 2023-10-14

**Authors:** Lottida Phondeth, Rajamanikkam Kamaraj, Julie Nilles, Johanna Weiss, Walter E. Haefeli, Petr Pávek, Dirk Theile

**Affiliations:** 1 Department of Clinical Pharmacology and Pharmacoepidemiology, Heidelberg University Hospital, Im Neuenheimer Feld 410, 69120 Heidelberg, Germany; 2 Department of Pharmacology and Toxicology, Faculty of Pharmacy, Charles University in Prague, Heyrovskeho 1203, 50005 Hradec Kralove, Czech Republic; 3 Boehringer Ingelheim Pharma GmbH & Co. KG, Birkendorfer Str. 65, 88397 Biberach an der Riss, Germany

**Keywords:** Rifampicin, Rifabutin, P-glycoprotein, Induction, Inhibition

## Abstract

Physiology-based pharmacokinetic modeling suggests that rifabutin can out-balance P-glycoprotein (P-gp) induction by concurrent P-gp inhibition. However, clinical or experimental evidence for this Janus-faced rifabutin effect is missing. Consequently, LS180 cells were exposed to a moderately (2 µM) and strongly (10 µM) P-gp-inducing concentration of rifampicin or rifabutin for 6 days. Cellular accumulation of the fluorescent P-gp substrate rhodamine 123 was evaluated using flow cytometry, either without (induction only) or with adding rifamycin drug to the cells during the rhodamine 123 efflux phase (induction + potential inhibition). Rhodamine 123 accumulation was decreased similarly by both drugs after 6-day exposure (2 µM: 55% residual fluorescence compared to non-induced cells, *P* < 0.01; 10 µM: 30% residual fluorescence compared to non-induced cells, *P* < 0.001), indicating P-gp induction. Rhodamine 123 influx transporters mRNA expressions were not affected, excluding off-target effects. Acute re-exposure to rifabutin, however, considerably re-increased rhodamine 123 accumulation (2 µM induction: re-increase by 55%, *P* < 0.01; 10 µM induction: 49% re-increase, *P* < 0.001), suggesting P-gp inhibition. In contrast, rifampicin only had weak effects (2 µM induction: no re-increase; 10 µM induction: 16% re-increase; *P* < 0.05). Molecular docking analysis eventually revealed that rifabutin has a higher binding affinity to the inhibitor binding site of P-gp than rifampicin (Δ*G* (kcal/mol) = −11.5 vs −5.3). Together, this study demonstrates that rifabutin can at least partly mask P-gp induction by P-gp inhibition, mediated by high affinity binding to the inhibitory site of P-gp.

**Supplementary Information:**

The online version contains supplementary material available at 10.1007/s00204-023-03618-w.

## Introduction

Rifampicin and rifabutin are antibiotics mostly used against mycobacterial infections. The safety of these compounds is hampered by their risk to lower the plasma levels of co-administered drugs by enhancing the intestinal expression and activity of proteins implicated in drug metabolism (e.g., cytochrome P-450 isoenzyme 3A4, CYP3A4) and drug transport (e.g., P-glycoprotein (P-gp), encoded by *ABCB1*). The risk of rifampicin and rifabutin to interact with co-administered drugs has been described and reviewed extensively. In general, rifabutin is considered less prone to affect the kinetics of victim drugs than rifampicin (Finch et al. 2002; Baciewicz et al. 2008), but the reasons for that difference remain largely unknown. Our recently published data revealed several new, yet unknown characteristics of rifabutin that at least in part can explain its weaker net perpetrator effects. First, a physiology-based pharmacokinetic model of the simultaneous administration of rifabutin and dolutegravir (P-gp substrate drug) suggested that rifabutin can enhance intestinal absorption of dolutegravir (high maximum plasma concentration, *C*_max_), even in the state of P-gp induction (repetitive daily intake of rifabutin) (Theile et al. 2023). That implies rifabutin might partly blunt P-gp induction by concurrent intestinal P-gp inhibition. Second, direct evidence for the proposed rifabutin-mediated P-gp inhibition was obtained in vitro, demonstrating that rifabutin indeed is a considerably more potent inhibitor of P-gp than rifampicin. However, these experiments used cell lines with murine *mdr1a/b* overexpression (mediated by long-term exposure to doxorubicin) (Theile et al. 2023) or genetically engineered cell lines with very high overexpression human P-gp (Nilles et al. 2023). In contrast, there is no data showing rifabutin-mediated P-gp induction and counteracting P-gp inhibition in the same model. Consequently, a P-gp induction model cell line was used to demonstrate this Janus-faced characteristic of rifabutin: LS180 cells were exposed to a moderately or strongly P-gp-inducing concentration of rifampicin or rifabutin for 6 days and subsequently evaluated for rhodamine 123 accumulation using flow cytometry, either without (induction only) or with adding rifamycin drug to the cells during the rhodamine 123 efflux phase (induction + potential inhibition). Finally, molecular docking simulations were performed in silico to analyze the molecular interaction of rifampicin and rifabutin with the P-gp protein structure.

## Materials and methods

### Materials

Cell culture flasks were obtained from Greiner (Frickenhausen, Germany). Dulbecco’s Modified Eagle’s Medium (DMEM) and fetal calf serum (FCS) were purchased from PAN-Biotech (Aidenbach, Germany). Phosphate-buffered saline (PBS), medium supplements for LS180 cell culture (glutamine, non-essential amino acids, penicillin/streptomycin), penicillin–streptomycin (100×), and zosuquidar were purchased from Sigma-Aldrich (Taufkirchen, Germany). Rifampicin was from Applichem (Darmstadt, Germany), rifabutin was purchased Toronto Research Chemicals (North York, Canada). Rhodamine 123 was purchased from CalBiochem (Darmstadt, Germany). The Absolute QPCR SYBR Green Mix was supplied by Abgene (Hamburg, Germany).

### Stock solutions

Rifampicin and rifabutin (100 mM stock solutions) were dissolved in DMSO and stored at −20 °C. The stock solutions were diluted with supplemented medium prior to the experiments. Rhodamine 123 (500 µM) and zosuquidar (10 µM) were dissolved in DMSO and stored at −20 °C. The DMSO concentrations in the assays did not exceed 0.1%.

### LS180 cell culture

LS180 cells originate from a human colon adenocarcinoma and are available at ATCC (Manassas, VA, USA). This cell line shows excellent functional inducibility of P-gp (Weiss et al. 2013; Nilles et al. 2023). LS180 cells were cultured under standard conditions with DMEM supplemented with 10% FCS, 2 mM glutamine, 100 U/mL penicillin, 100 µg/mL streptomycin sulphate, and 0.1 mM non-essential amino acids.

### P-gp induction and P-gp inhibition upon acute drug re-exposure

**Fig. 1:**
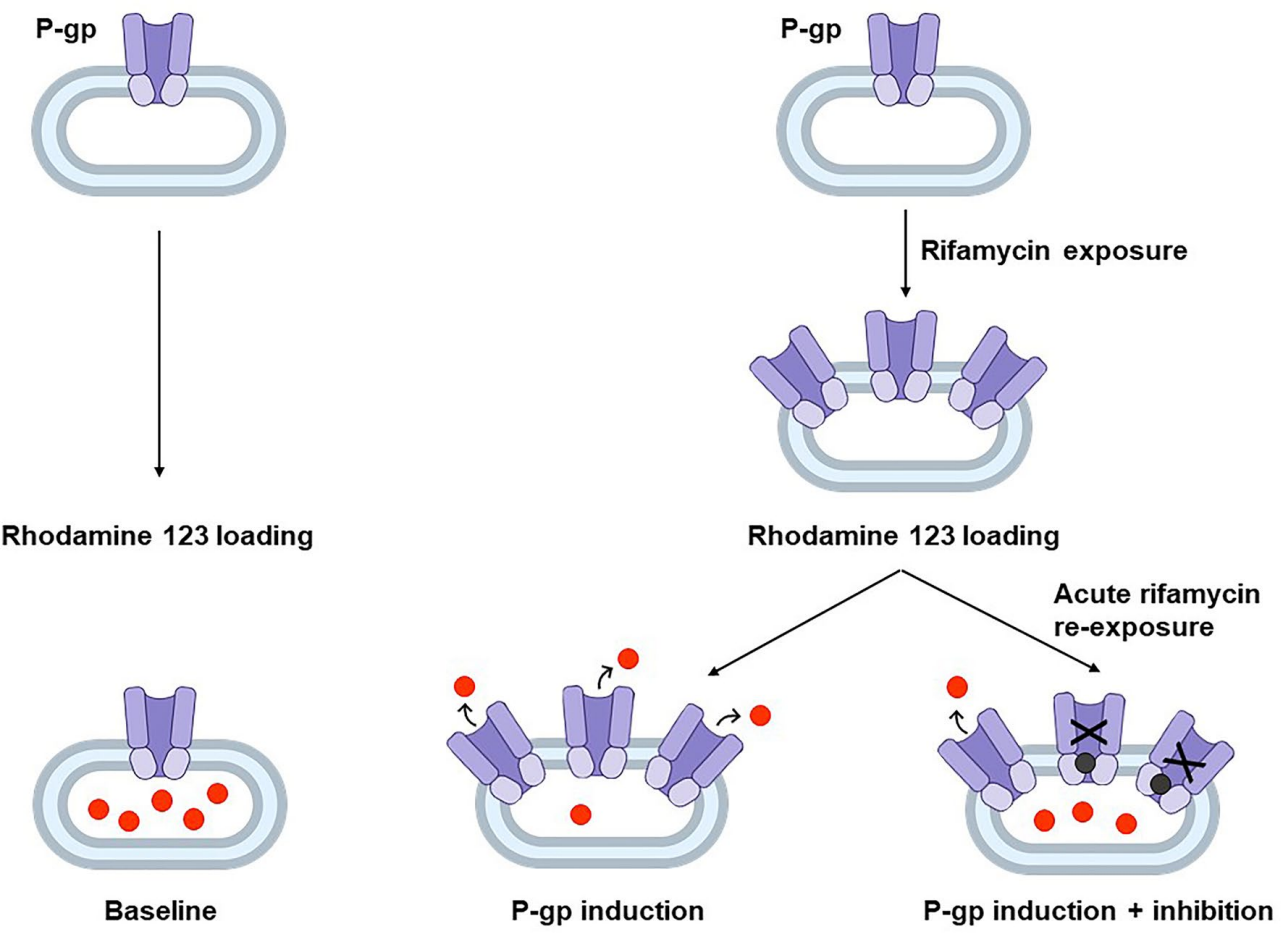
Experimental design and hypothesized outcome: LS180 cells were left untreated (left) or exposed to 2 or 10 µM of rifampicin or rifabutin for 6 days (right). Subsequently, cells were washed and loaded with rhodamine 123, a fluorescent P-gp substrate (red dots). During the following rhodamine 123 efflux phase, cells were either left untreated (P-gp induction, low rhodamine 123 accumulation) or acutely re-exposed to different concentrations of the rifamycin drugs or zosuquidar (black dots) (P-gp induction + P-gp inhibition, re-increasing rhodamine 123 accumulation) (color figure online)

To assess P-gp activity in LS180 cells, the previously published experimental approach (Weiss et al. 2008; Theile et al. 2013; Nilles et al. 2023) was used, with some modifications: LS180 cells were cultured in standard cell culture flasks and exposed to 2 or 10 µM of rifampicin or rifabutin for six consecutive days, conditions known to cause moderate or strong functional P-gp induction (Nilles et al. 2023). Cells were detached, washed, distributed to 1.2 × 10^6^ cells per tube and loaded with rhodamine 123 (0.4 µM in RPMI/2% FCS medium) for 30 min at 37 °C with continuous shaking and being protected from light. After this loading step, the cells were centrifuged (5 min; 1000*g*; 4 °C), washed with ice-cold RPMI/2% FCS, and re-exposed to the rifamycin drugs (0.5–50 µM), zosuquidar (10 µM), or left untreated (RPMI/2% FCS without drug) for 50 min at 37 °C with continuous shaking and being protected from light (rhodamine 123 efflux phase). After this efflux phase, the cells were washed with ice-cold PBS/2% FCS, resuspended in 300 µL PBS/2% FCS and transferred into the flow cytometry sample tubes. Intracellular rhodamine 123 fluorescence was determined using the MACSQuant analyzer 10 (Miltenyi Biotec, Bergisch Gladbach, Germany). In each sample, 30,000 gated cells were counted and the median rhodamine 123 fluorescence was recorded as the primary read-out. Experiments were performed in three independent biological replicates and the mean rhodamine 123 fluorescence is reported as the final read-out. The design of the experiments is shown in Fig. 1.

### Quenching control experiments

To assess potential fluorescence interferences between rhodamine 123 and the rifamycin drugs or zosuquidar, 50 µL rhodamine 123 solutions (0.1 µM, dissolved in PBS with 2% FCS) were mixed with 50 µL rifamycin drugs (0.5–50 µM in PBS with 2% FCS) or zosuquidar (1 or 10 µM in PBS with 2% FCS) in a black 96-well plate and were subsequently incubated light-protected for 30 min at 37 °C. Rhodamine 123 fluorescence was recorded at an excitation wave length of 485 nm and emission wave length of 535 nm using a SpectraMax iD3 plate reader from Molecular Devices (Ismaning, Germany).

### Impact of rifampicin and rifabutin on rhodamine 123 influx transporters mRNA expression

In our recently published experiments (Nilles et al. 2023), LS180 cells had identically been exposed to rifampicin or rifabutin. Accordingly, the already synthesized cDNA was used to evaluate the drug effects on the mRNA expression levels of two important rhodamine 123 influx transporters, solute carrier family 22 member 1 (*SLC22A1*, encoding organic cation transporter 1, OCT1) and solute carrier organic anion transporter family member 1A2 (*SLCO1A2*, encoding organic anion transporting polypeptide 1A2, OATP1A2). Expression levels were quantified by real-time reverse transcription (RT) polymerase chain reaction (PCR) with a LightCycler^®^ 480 (Roche Applied Science, Mannheim, Germany) (Albermann et al. 2005; Weiss et al. 2011) and Quantitect primer kits (Qiagen). PCR conditions have been published previously (Albermann et al. 2005; König et al. 2010). Among a set of six housekeeping genes tested, glucuronidase beta had been identified the most stable (Nilles et al. 2023) and was consequently used again for normalization. Data were analyzed as described previously (Albermann et al. 2005). Experiments had been performed in four independent biological replicates with technical duplicates (PCR runs) for each concentration.

### Molecular docking

Docking studies were performed using the Schrödinger Maestro 13.3 software package. The conformation of compounds was generated and energy-minimized by the LigPrep tool using force field OPLS4. The 3D crystal structure of P-gp (PDB code: 6QEE and 6QEX) was obtained from the RCSB Protein Data Bank (https://www.rcsb.org/) (Alam et al. 2019). The obtained receptor was prepared (involving optimization, charge calculation, deletion of co-crystal ligand, and addition of hydrogen, etc.) with Protein Preparation Workflow. The GLIDE tool was employed to dock ligands at the substrate and inhibitor binding sites with a high degree of accuracy (Brožová et al. 2023). The proposed binding modes were visualized using a combination of software tools, including the Maestro workspace, and PyMOL to generate high-quality images. The obtained docking results of the P-gp/drug complexes were used to predict the values of the binding affinity (Δ*G*) (kcal/mol) using the PROtein binding enerGY prediction (PRODIGY-LIGAND) online server tool (Vangone et al. 2019).

### Statistics

For each induction effect (2, 10 μM), three independent biological replicates were evaluated. For data computation, the background fluorescence signal (untreated and unstained LS180 cells) was subtracted from all samples. Afterwards, the rhodamine 123 signals of all other samples were normalized to the signal from untreated LS180 cells (baseline fluorescence, set to 1.0). The statistical evaluation was performed using GraphPad Prism version 9.5.1. The untreated cell control was compared to the induced cells by the Student’s *t*-test. The comparison of fluorescence in induced cells without re-exposure (induction only) to cells being acutely re-exposed to rifamycin drugs or zosuquidar of specific concentrations was performed using an ANOVA with non-parametric Kruskal–Wallis test. The comparison between the obtained plateau of the sigmoidal concentration–response-curve (*E*_max_) and the induced cells were performed using the Student’s *t*-test. The percentual increase in rhodamine 123 fluorescence upon acute drug re-exposure was calculated for each triplicate according to the following formula: ((relative *E*_max_ / relative induction effect) − 1) × 100%. The resulting mean ± SEM percentual increase of rhodamine 123 fluorescence is reported. Percentual increases mediated by rifampicin and rifabutin were compared by Student’s *t*-test. Impact of rifamycin exposure on mRNA expression levels was evaluated by an ANOVA with non-parametric Kruskal–Wallis test. *P*-values < 0.05 were considered significant.

## Results

### P-gp induction and P-gp inhibition upon acute drug re-exposure

**Fig. 2:**
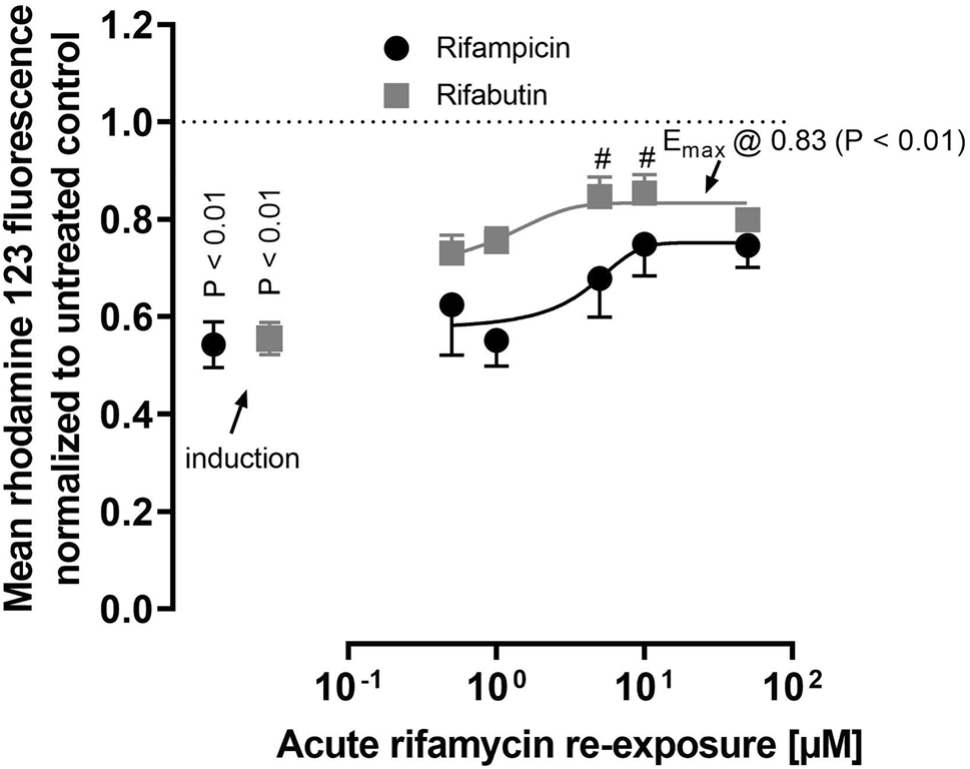
Mean rhodamine 123 fluorescence in LS180 cells treated with 2 µM rifampicin (black circles) or rifabutin (gray squares) for 6 days and subsequently re-exposed to the respective rifamycin drug during the rhodamine 123 efflux phase, normalized to untreated control cells (non-induced, no re-exposure; dotted line). Data shown is the mean ± SEM of triplicates. The comparison between the non-induced cell controls and induced cells was performed with the Student’s *t*-test. Rifamycin concentrations that significantly re-increased the fluorescence signal were evaluated by ANOVA with non-parametric Kruskal–Wallis test and are indicated by hash key symbols (#*P*-value < 0.05). The concentration–effect curve was fitted according to a four parameter-logistic equation (= *E*_max_ model; variable slope). The resulting plateau (*E*_max_) values were compared to induced cells by Student’s *t*-test. A *P*-value < 0.05 was considered significant

Cells having been initially exposed to 2 μM rifampicin or rifabutin for 6 days showed significant mean cellular fluorescence reductions to 0.55% (rifampicin treatment; *P* = 0.007) and 0.56% (rifabutin treatment; *P* = 0.001), compared to untreated cell controls (Fig. 2; Table S1). These induction effects did not differ (*P* = 0.85). Re-exposing the cells to rifampicin during the rhodamine 123 efflux phase did not re-increase the cellular fluorescence. On the contrary, 5 μM (*P* = 0.047) and 10 μM rifabutin (*P* = 0.014) significantly re-increased the fluorescence. The resulting *E*_max_ of the acute rifabutin re-exposure (0.83%; *P* < 0.01) was 49% higher compared to the fluorescence in induced cells (Fig. 2; Table S1). The positive control for P-gp inhibition zosuquidar (10 µM) re-enhanced the signals back to 0.75 (rifampicin treatment) and 0.86 (rifabutin treatment), albeit without statistical significance compared to induced cells (data not shown).

**Fig. 3:**
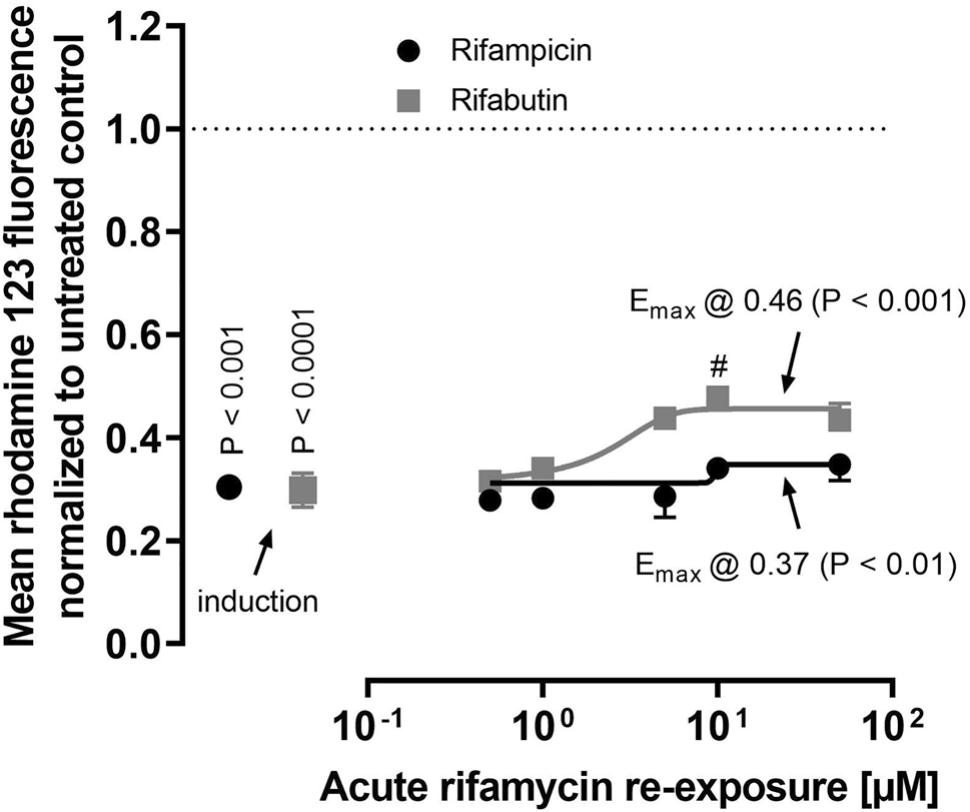
Mean rhodamine 123 fluorescence in LS180 cells treated with 10 µM rifampicin (black circles) or rifabutin (gray squares) for 6 days and subsequently re-exposed to the respective rifamycin drug during the rhodamine 123 efflux phase, normalized to untreated control cells (non-induced, no re-exposure; dotted line). Data shown is the mean ± SEM of triplicates. The comparison between the non-induced cell controls and induced cells was performed with the Student’s *t*-test. Rifamycin concentrations that significantly re-increased the fluorescence signal were evaluated by ANOVA with non-parametric Kruskal–Wallis test and are indicated by hash key symbols (#*P*-value < 0.05). The concentration–effect curve was fitted according to a four parameter-logistic equation (= *E*_max_ model; variable slope). The resulting plateau (*E*_max_) values were compared to induced cells by Student’s *t*-test. A *P*-value < 0.05 was considered significant

Six-day exposure to 10 μM rifampicin or rifabutin significantly reduced mean rhodamine 123 fluorescence to 0.31% (rifampicin treatment; *P* = 0.001) and 0.29% (rifabutin treatment; *P* < 0.0001), indicating considerable P-gp induction (Fig. 3, Table S1). Again, induction effects between rifampicin and rifabutin did not differ (*P* = 0.23). None of the acute rifampicin re-exposure concentrations re-increased rhodamine 123 fluorescence, suggesting lack of P-gp inhibition. On the other hand, 10 μM rifabutin significantly re-increased the fluorescence compared to the induced cells (*P* = 0.005). Both acute re-exposures to variable concentrations of rifampicin (*P* = 0.002) and rifabutin (*P* = 0.0004) reached a plateau of rhodamine 123 fluorescence being significantly higher than in the induced cells (Fig. 3, Table S1). However, the mean percentual increase of rhodamine 123 fluorescence mediated by acute rifabutin re-exposure (55%) was higher than the re-increase mediated by rifampicin (16%; *P* = 0.047). Zosuquidar caused a return of fluorescence signals to 0.466 for both rifamycin inductions, being insignificantly different to the rhodamine 123 values in induced cells. Fluorescence interference of compounds was excluded by quenching assays (data not shown). The data on rifamycin induction and acute re-exposure are detailed in supplementary Table S1.

### Impact of rifampicin and rifabutin on rhodamine 123 influx transporters mRNA expression

Six-day exposure of LS180 cells to rifampicin (0.1–50 µM) or rifabutin (0.1–20 µM) did not change mRNA expression levels of *SLC22A1*. *SLCO1A2* was too low expressed (crossing-point beyond 34–35 cycles) before and after rifamycin exposure to be properly quantified (data not shown).

### Molecular docking

**Fig. 4:**
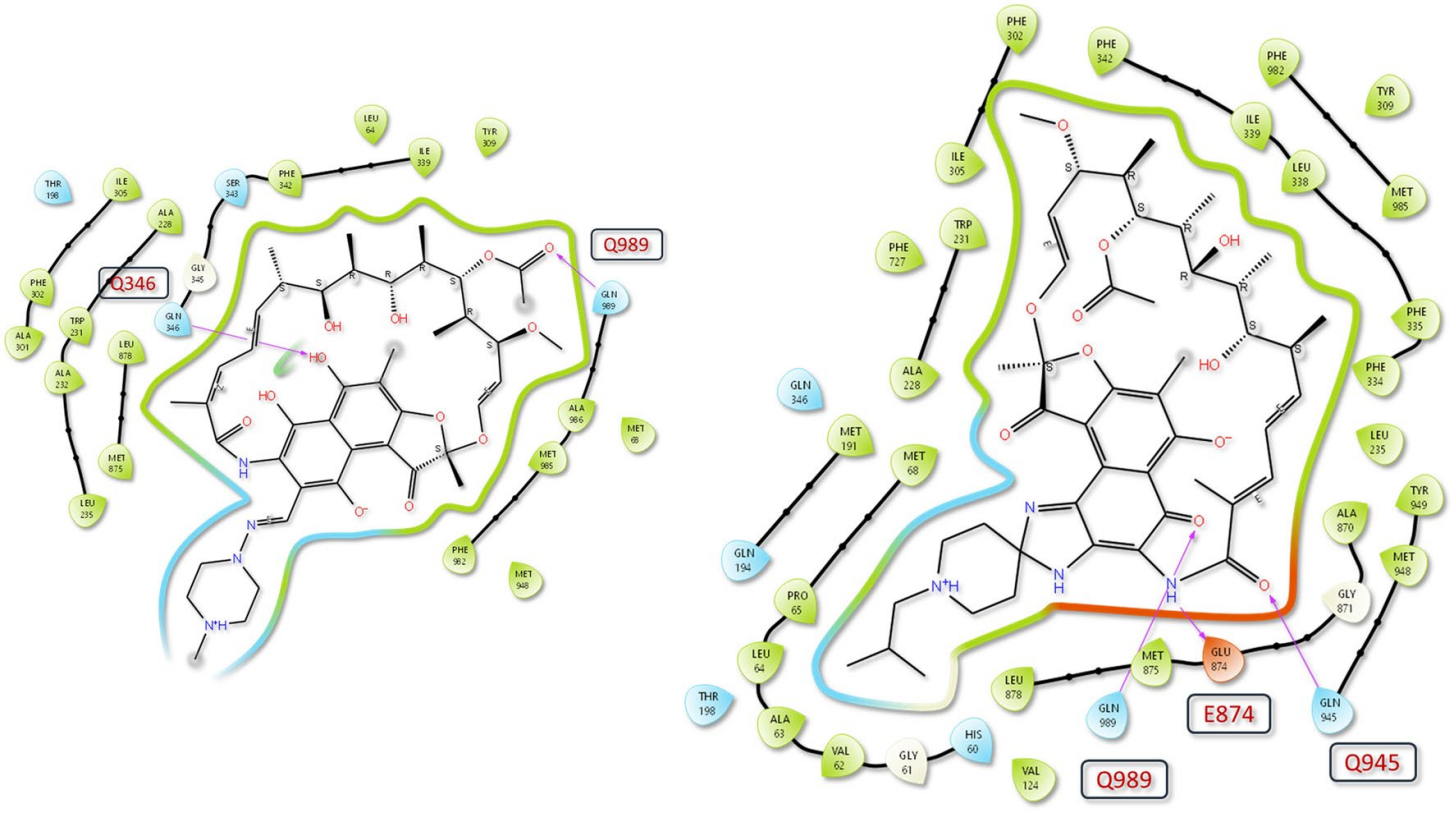
Two-dimensional interactions of rifampicin and rifabutin with P-gp at the inhibitor binding site. The figure shows the schematic diagrams of the molecular interactions between rifampicin (left) and rifabutin (right) with the residues of P-gp that form the inhibitor binding site (M-site). The hydrogen bonds are represented by pink arrows (color figure online)

**Fig. 5:**
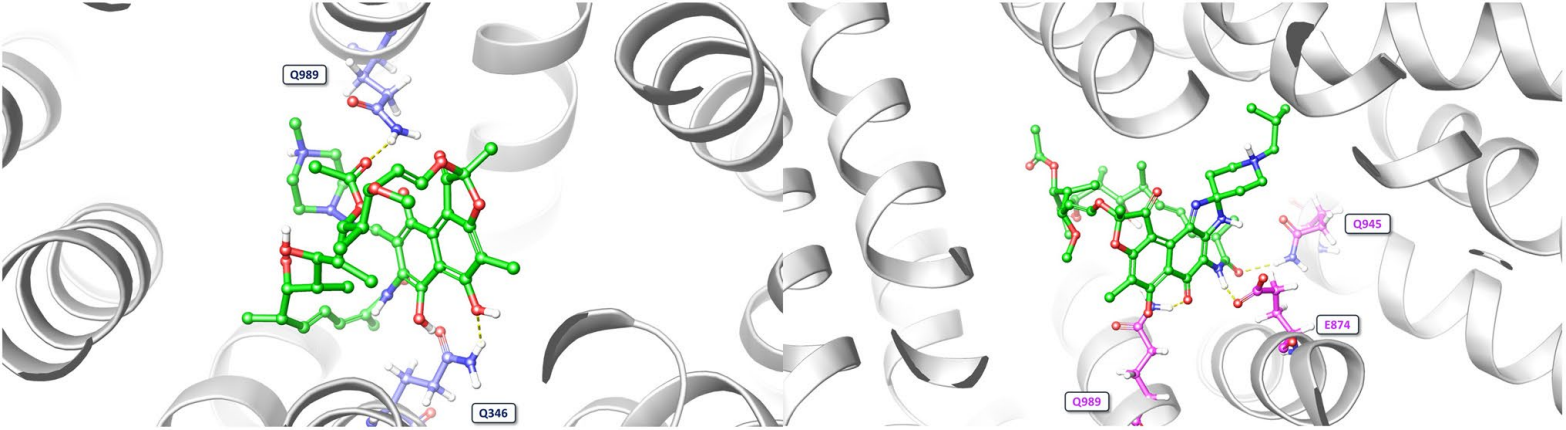
Three-dimensional interactions of rifampicin and rifabutin with P-gp at the inhibitor binding site. The figure shows the molecular docking models of rifampicin (left) and rifabutin (right) with the residues of P-gp that form the inhibitor binding site (M-site). The hydrogen bonds are represented by grey dashed lines. The residues that interact with rifampicin are colored in blue, and the residues that interact with rifabutin are colored in pink. The ligands are shown in green, and the residue names are indicated in boxes (color figure online)

Molecular docking studies showed that rifampicin and rifabutin have different binding affinities and interactions with P-gp at the inhibitor binding site (M-site) and the substrate binding site. The 2D/3D ligand diagrams of the interactions are shown in Figs. 4 and 5, respectively. At the M-site, rifampicin formed hydrogen bond interactions with Q989 and Q346, and hydrophobic interactions with other residues of P-gp. However, the docking score of rifampicin was low (−2.73 kcal/mol; Table 1), suggesting low inhibitory action. In contrast, rifabutin formed hydrogen bond interactions with Q989, Q945, and E874, more hydrophobic interactions than rifampicin, and eventually had a high docking score (−6.36 kcal/mol), suggesting strong binding to the inhibitory M-site. Moreover, rifabutin had a lower free energy of binding (Δ*G*) (−11.51 kcal/mol) than rifampicin (−5.39 kcal/mol). As a positive control for P-gp inhibition, zosuquidar was evaluated as well, showing a similar Δ*G* as rifabutin (−10.67 kcal/mol).

**Table 1:** GLIDE docking scores and binding affinities with P-gp

Compound	Glide docking score (kcal/mol)		Binding affinity
	Substrate binding site (6QEX)	Inhibitor binding site (6QEE)	Δ*G* (kcal/mol) (6QEE)
Paclitaxel	−10.07	−7.38	–
Rifabutin	−8.47	−6.36	−11.51
Rifampicin	−7.68	−2.73	−5.39
Zosuquidar	−7.86	−9.47	−10.67

**Fig. 6:**
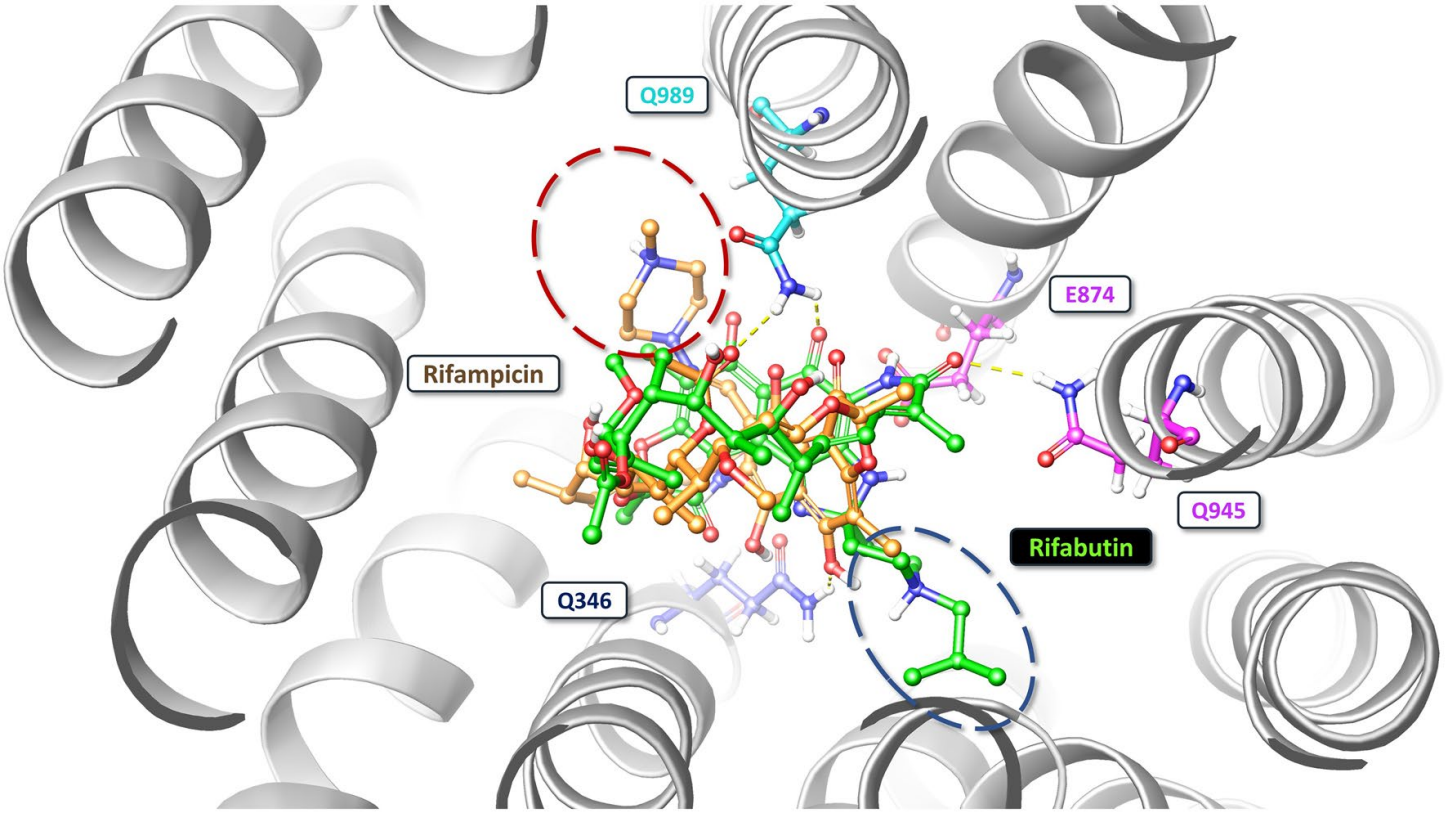
Comparison of rifampicin and rifabutin binding modes with P-gp. The figure shows the superimposed image of the molecular docking models of rifampicin (substrate) and rifabutin (inhibitor) with P-gp. Rifabutin is shown in green and rifampicin is shown in beige. The blue dotted ring highlights the extension of the spiro piperidine moiety of rifabutin, which interacts with the inhibitor binding site (M-site). The red dotted circle marks the piperazine moiety of rifampicin, which is located at the substrate binding site. The cyan-colored residues are common for both ligands (color figure online)

At the substrate binding site, both rifampicin and rifabutin had good interactions with P-gp, shown by their docking scores (−7.68 and −8.47 kcal/mol, respectively). The well-known P-gp substrate paclitaxel had the highest docking score (−10.07 kcal/mol). Interestingly, both rifampicin and rifabutin bound to the same site, but with different orientations (Fig. 6). Rifabutin had a cross interaction between the substrate and inhibitor binding sites through its spiro piperidine moiety, while rifampicin did not show such a cross interaction (Fig. 6).

## Discussion

The objective of this study was to investigate whether rifamycin-mediated P-gp induction can functionally be blunted by concurrent drug-mediated P-gp inhibition. Accordingly, rhodamine 123 accumulation in the induced cells was evaluated, either left untreated or being acutely re-exposed to different rifampicin or rifabutin concentrations during the rhodamine 123 efflux phase. The data obtained revealed several important findings: First, this study complements and supports our previous observations, demonstrating that both rifamycin drugs can considerably increase P-gp activity after 6 days of exposure (Nilles et al. 2023). Second, rifabutin but not rifampicin seems capable of partly out-balancing a prior P-gp induction by concurrent P-gp inhibition. After 6 days of exposure to 10 μM rifampicin, none of the acute rifampicin exposure concentrations significantly re-increased the rhodamine 123 fluorescence and the estimated *E*_max_ of out-balance was only 16% higher than the fluorescence in induced cells without acute re-exposure. In contrast, after long-term exposure to 10 μM rifabutin, 10 μM acute rifabutin re-exposure significantly re-increased rhodamine 123 fluorescence and the estimated *E*_max_ plateau was 55% higher than in induced cells without acute re-exposure. The results obtained from the induction phase with 2 μM rifamycin drug further substantiate the assumption that rifabutin indeed is the stronger P-gp inhibitor and ‘compensator’. Again, even though 2 μM rifampicin and rifabutin decreased rhodamine 123 accumulation similarly, only re-exposure to rifabutin (5, 10 μM) re-increased the fluorescence signals, with an estimated *E*_max_ of 49% higher than in induced cells. Together, this data suggest that rifabutin can at least in part blunt P-gp induction by concurrent P-gp inhibition.

Because P-gp activity was assessed by rhodamine 123 accumulation, potential off-target effects or by-stander effects must be considered, and the validity of the experimental approach should be questioned. First, did the reduction of rhodamine 123 fluorescence truly result from P-gp induction or are there other transporters potentially contributing to the observed effects? Indeed, rhodamine 123 is additionally extruded by the multidrug resistance-associated protein 2 (MRP2) (Jouan et al. 2014; Twentyman et al. 1994) and the breast cancer resistance protein (BCRP) (Jouan et al. 2014). However, we (Weiss et al. 2011) and others (Gupta et al. 2008) have shown that rifampicin does not relevantly affect the expression of these efflux transporters in LS180 cells, supporting the assumption that P-gp was mainly affected by long-term rifamycin treatment. Alternatively, reduced rhodamine 123 fluorescence after long-term rifamycin treatment could have resulted from suppression of rhodamine 123 influx transporter expression. However, rifampicin is known not to down-regulate (Gupta et al. 2008) the rhodamine 123 influx transporter *SLCO1A2*/OATP1A2 (Forster et al. 2012; Jouan et al. 2014). In our set of experiments, this transporter’s expression was so low (crossing point beyond 34–35 cycles) that it could not be assessed quantitatively, indicating no relevant expression in LS180 cells. The expression of another important rhodamine 123 influx transporter in LS180 cells (*SLC22A1*, OCT1) (Jouan et al. 2014) was not affected. Moreover, known inhibitory effects of rifabutin on OCT1 (Parvez et al. 2016) can largely be ruled out because cells were re-exposed to the rifamycin drugs *after* rhodamine 123 loading and thorough washing steps. Finally, chemo-physical interaction of rifamycin drugs with rhodamine 123 was excluded by quenching assays. Together, functional P-gp induction very likely was the most important factor that contributed to the observed reduction of rhodamine 123 accumulation following the long-term exposure to rifampicin or rifabutin. Second, why was it not possible to completely out-balance P-gp induction by re-exposing the induced cells to rifampicin, rifabutin, or zosuquidar? The latter almost completely fills the P-gp substrate pocket (Alam et al. 2018), traps and inhibits the ATP-hydrolysis site (Alam et al. 2019), and thus is considered a highly potent, non-competitive, and selective inhibitor of P-gp (Alam et al. 2018, 2019; Dantzig et al. 2001). Again, known inhibitory effects of rifampicin or zosuquidar on OATP1A2 (Amor et al. 2018; Franke et al. 2009) seem unlikely given the separate steps of rhodamine 123 loading, washing, and re-exposure. Regarding OCT1, previous studies have already indicated that rifampicin (Parvez et al. 2016) and zosuquidar (Nies et al. 2008) do not inhibit it, when [^3^H]N-methyl-4-phenylpylidinium acetate or berberine were used as OCT1 substrates. One hundred μM rifabutin in fact inhibits OCT1 by 50% (Parvez et al. 2016), but in our experiments, re-exposure concentrations never exceeded 50 μM. Together, it remains open why fluorescence did not completely return to baseline levels. Despite these uncertainties, the data suggest that rifabutin can partly out-balance P-gp induction by inhibitory effects. To further substantiate this assumption and to estimate the mode of inhibitory action, molecular docking studies were performed. The obtained findings suggest that rifabutin binds P-gp more strongly than rifampicin at the inhibitor binding sites. Structural analysis and binding free energy (Δ*G*) scores support this statement (−11.51 vs −10.67 kcal/mol). The main reason for this difference is that rifabutin has a tight hydrogen bond with E874, a key residue in the inhibitor access tunnel of the tenth transmembrane domain (TM10) (Smolinski et al. 2021; Ferreira et al. 2013). Additionally, the spiro piperidine group of rifabutin covers both the substrate and inhibitor sites, potentially changing P-gp’s shape and function in TM10 and eventually increasing its inhibition (Alam et al. 2019; Nosol et al. 2020).

To date, there are no clinical data confirming P-gp inhibition by rifabutin. In contrast, some trials have, however, suggested P-gp inhibition by rifampicin. For instance, in a study conducted by Reitman and co-workers, digoxin area under the time–concentration-curve (AUC) and *C*_max_ were increased by 46% and 49% when digoxin had been administered 1 h after the last rifampicin dose of a 4-week induction phase with 600 mg/day rifampicin. The authors explained this increase of AUC and *C*_max_ by an acute inhibitory effect of rifampicin on intestinal P-gp and additionally showed in vitro that rifampicin inhibits P-gp with an IC_50_ of 169 ± 18 µM. In contrast, when digoxin had been administered 1 week after rifampicin discontinuation, the AUC and *C*_max_ were decreased to approximately 30%, representing intestinal P-gp induction (Reitman et al. 2011). Similar observations have been made with fexofenadine (another P-gp substrate), but this data might have been influenced by concurrent interference of rifampicin with OATP1B1, OATP1B3, and OATP2B1 (Kusuhara et al. 2013; European Medicines Agency ICH guideline M12 on drug interaction studies). In summary, there is some evidence that rifampicin can inhibit P-gp. However, we expect rifabutin to cause considerably stronger P-gp inhibition given the data presented here and published previously (Theile et al. 2023; Nilles et al. 2023). Accordingly, prospective clinical trials should compare the induction effects and acute P-gp inhibitory actions of rifampicin versus rifabutin, evaluated by proposed P-gp marker compounds such as dabigatran etexilate (Lutz et al. 2018a, b; European Medicines Agency 2023).

## Conclusion

To conclude, this study demonstrated for the first time in vitro and in silico that rifabutin-inflicted P-gp induction can be functionally blunted by rifabutin through concurrent P-gp inhibition, likely mediated by strong binding to key residues of the inhibitory M-site of P-gp. This data not only underline previous experimental or pharmacometric findings but also advocate for prospective clinical trials that evaluate this Janus-faced characteristics of rifabutin.

### Supplementary Information

Below is the link to the electronic supplementary material.

Supplementary file1 (DOCX 17 KB)

## Data Availability

Original data are available upon reasonable request.
