# Supplementary material for: Rifabutin but not rifampicin can partly out-balance P-glycoprotein induction by concurrent P-glycoprotein inhibition through high affinity binding to the inhibitory site
Source: Arch Toxicol. 2023 Oct 14;98(1):223–31. doi: 10.1007/s00204-023-03618-w (PMC10761502; doi:10.1007/s00204-023-03618-w)
Supplement: Supplementary file 1 — Supplementary file1 (DOCX 17 KB) [file 204_2023_3618_MOESM1_ESM.docx]

**Rifabutin but not rifampicin can partly out-balance P-glycoprotein induction by concurrent P-glycoprotein inhibition through high affinity binding to the inhibitory site**

Lottida Phondeth^1*^, Rajamanikkam Kamaraj^2*^, Julie Nilles^1,3^, Johanna Weiss^1^, Walter E. Haefeli^1^, Petr Pávek^2^, Dirk Theile^1^

**Supplementary material:**

Supplementary Table S1: Summary of obtained data.

|  | **Induction effect^a^** | | **Significant out-balancing concentration^b^** | | **Maximum out-balance^c^** | | **Percentual maximum out-balance^d^** | |
| --- | --- | --- | --- | --- | --- | --- | --- | --- |
|  | Rifampicin | Rifabutin | Rifampicin | Rifabutin | Rifampicin | Rifabutin | Rifampicin | Rifabutin |
| **2 µM** | 0.55 ± 0.041  P = 0.007 | 0.56 ± 0.017  P = 0.001 | X | 5 µM  P = 0.047;  10 µM  P = 0.014 | X | 0.83 ± 0.026  P = 0.0020 | X | 49.0 % ± 1.9 % |
|  | X | |  |  |  |  | X | |
| **10 µM** | 0.31 ± 0.0026  P = 0.001 | 0.29 ± 0.010  P < 0.0001 | X | 10 µM  P = 0.005 | 0.37 ± 0.0036  P = 0.002 | 0.46 ± 0.0084  P = 0.0004 | 16.2 % ± 1.7 % | 55.4 % ± 7.5 % |
|  | X | |  |  |  |  | P = 0.047 | |
| 1. Rhodamine 123 fluorescence after six days of exposure compared to non-induced cells. 2. Distinct re-exposure concentrations that significantly re-increased rhodamine 123 fluorescence compared to induced cells. 3. Estimated maximum rhodamine 123 fluorescence after drug re-exposure compared to non-induced cells. 4. Percentual re-increase of rhodamine 123 fluorescence in re-exposed cells compared to induced cells. | | | | | | | | |

The induction effect is the mean ± S.E.M. relative rhodamine 123 fluorescence in induced cells normalized to untreated controls (Student’s T-test). The maximum out-balance values represent the estimated relative E_max_ of the fitted concentration-response curve and was compared to the induction effect (Student’s t-test). The distinct concentrations having significantly re-increased relative rhodamine 123 fluorescence were evaluated by ANOVA with the Kruskal-Wallis test. The mean percentual maximum out-balance was calculated by dividing the relative E_max_ by the induction effect. Percentual re-increases by rifampicin and rifabutin were compared by Student’s T-test. A P value < 0.05 was considered significant. X indicates non-significant results or non-significant differences between sub-columns.
